# Women with Gaucher Disease

**DOI:** 10.3390/biomedicines12030579

**Published:** 2024-03-05

**Authors:** Maria del Mar Meijon-Ortigueira, Isabel Solares, Cecilia Muñoz-Delgado, Sinziana Stanescu, Marta Morado, Cristina Pascual-Izquierdo, Lucía Villalon Blanco, Amaya Belanger Quintana, Covadonga Pérez Menéndez-Conde, Montserrat Morales-Conejo, Jesús Villarrubia-Espinosa

**Affiliations:** 1 Department of Hematology, Hospital Universitario Ramón y Cajal-IRYCIS, 28034 Madrid, Spain; 2 Department of Internal Medicine, Clínica Universidad de Navarra, 28027 Madrid, Spain; 3 Department of Internal Medicine, Hospital Quirónsalud Sur, 28922 Madrid, Spain; 4 Pediatric Metabolic Unit, Hospital Universitario Ramón y Cajal, European Reference Center (MetabERN), 28034 Madrid, Spain; 5 Department of Hematology, Hospital Universitario La Paz, 28046 Madrid, Spain; 6 Department of Hematology, Hospital Universitario Gregorio Marañon, 28007 Madrid, Spain; 7 Department of Hematology, Hospital Universitario Fundación Alcorcón, 28922 Madrid, Spain; 8 Centro de Investigación Biomédica en Red de Enfermedades Raras (CIBERER), 28029 Madrid, Spain; 9 Pharmacy Department, Ramón y Cajal Hospital, IRYCIS, 28034 Madrid, Spain; 10 Department of Internal Medicine, Hospital Universitario 12 de Octubre, 28041 Madrid, Spain

**Keywords:** Gaucher disease, women, enzyme-replacement therapy (ERT), substrate-reduction therapy (SRT), lysosomal-storage disorder

## Abstract

Gaucher disease is an inherited disorder in which there is a deficiency of the enzyme glucocerebrosidase, which leads to the accumulation of glucosylceramide. Although much scientific evidence is now available, there is still limited data on the impact on the different life stages of women with this disease. Among other alterations, a delay in menarche has been described, although it has not been related to fertility problems. Menorrhagia is relatively frequent, being related to the presence of thrombocytopenia, thrombocytopathies or coagulation disorders. On the other hand, pregnancy planning is an increasingly frequent concern. All patients should undergo genetic counseling, and it is important to monitor the appearance or worsening of organomegaly, bone and hematologic abnormalities to establish clinical and therapeutic recommendations. Management during the puerperium will depend on the evolution of gestation, and, during the lactation period, the potential appearance of bone complications should be assessed. An early onset of menopause, compared to the general population, has also been described, which may accelerate the development of osteopenia. Finally, although the usual screening protocols for neoplasms are currently being performed, it is recommended to watch for early signs of liver or renal neoplasms when examining the results of imaging tests performed during evaluations for this disease.

## 1. Introduction

Gaucher disease (GD) is a rare hereditary disease caused by mutations in the *GBA1* gene, which encodes the enzyme glucocerebrosidase, resulting in a deficiency in this enzyme [1]. This deficiency leads to the accumulation of the substrate, glucosylceramide, mainly in macrophages of the liver, spleen, and bone marrow.

GD has been classified into three clinical phenotypes based on age of onset, progression, and neurological involvement. Type 1 GD is the non-neuronopathic form and is characterized by the presence of hematological abnormalities (such as anemia and thrombocytopenia), hepatosplenomegaly and bone involvement. Lung involvement is observed less frequently. Type 2 GD is the acute neuronopathic form and is characterized by significant involvement of the central nervous system at an early age, with a high mortality rate in childhood. Type 3 is also associated with neurological involvement, but with a less severe course than Type 2 and with a better prognosis. GD is also associated with other comorbidities, such as Parkinson’s disease or the increase in hematology neoplasm, especially multiple myeloma [2].

Regarding treatment, we currently have two types of therapy: enzyme-replacement therapy (ERT), which consists of the intravenous administration of the active enzyme, currently available on the Spanish market as Imiglucerase (Cerezyme^®^) and Velaglucerase alfa (VPRIV^®^), and substrate-reduction therapy (SRT), which consists of the inhibition of the enzyme glucosylceramide synthetase. There are two products available on the Spanish market, one in the first line of treatment, eliglustat, and the other in the second line, miglustat. Miglustat is a second-line treatment indicated for the small group of patients with GD1 in whom first-line treatment is not medically possible. In contrast, eliglustat is a first-line treatment, like ERT, and is applicable to all patients with GD1 who are not ultra-rapid metabolizers of cytochrome CYP2D6 [3]. These treatments have increased the survival and the quality of life of the patients [4].

Although there is an increasing amount of knowledge on the clinical characteristics of GD, there is little evidence on the behavior of the disease symptomatology in the different stages of a woman’s life, as well as on its treatment.

## 2. Childhood and Adolescence

### 2.1. Changes in Growth

Growth retardation in GD is characteristic [5,6], with some series observing an incidence of up to 50% of severe linear growth retardation in untreated patients during the first two decades of life [7]. The etiology is probably multifactorial: medullary infiltration via Gaucher cells, the production of inflammatory cytokines, the increased resting energy expenditure associated with the disease itself, or the deficiency of insulin-like growth factors in the context of liver involvement. These findings are more common in patients with severe disease but there are no differences based on sex [8].

### 2.2. Puberty

A delay in puberty has been described in patients with GD, with a frequency of up to 60% in some series [5]. This is more frequent in patients with severe disease [8]. Delayed menarche has been described in women with GD compared to other family members and the general population [9]. Despite the pubertal delay, in all patients, sexual maturity is reached and no secondary alterations in fertility are observed [10,11].

### 2.3. Menstruation

Heavy menstrual bleeding or menorrhagia is a major problem in patients with GD. Excessive menstrual bleeding has been described in 73% of patients. This menorrhagia is not related to the severity of the GD but to the presence of thrombocytopenia, altered platelet function or coagulation in the GD [11,12]. In the Ashkenazi population, this is added with the frequent deficiency of factor XI [9,11]. On the contrary, the average duration of menstruation is within normal limits [11].

Menorrhagia can aggravate preexisting anemia in GD patients. In this context, treatment with iron supplementation and, in some cases, an antifibrinolytic agent, may be necessary. Treatment with oral contraceptives is considered a good therapeutic option for young patients with menorrhagia [4]. Increased menstrual bleeding associated with intrauterine devices (IUDs) has been reported in up to 50% of patients, especially if associated with severe disease [9].

### 2.4. Psychosocial Sphere

Special consideration must be given to the psychosocial sphere in children and adolescents with GD. As with other chronic illnesses, it can be difficult to acknowledge the disease, and they may experience feelings of anger, denial, fear, insecurity and isolation [13]. Growth retardation and delayed puberty can influence body image and social functioning, resulting in psychosexual problems, as well as low self-esteem. The problems derived from bone involvement can prevent them from carrying out common physical activities in children, which worsens the feelings already described. In addition, chronic pain can affect school performance. However, there are no differences regarding sex in this scenario.

## 3. Adulthood

### 3.1. Contraceptive Methods

Reproductive planning is an increasingly frequent concern in women with GD. Although there is little information to help us with the decision-making process about the ideal contraceptive method in these patients, recent registries describe frequent management with oral contraceptives or surgical methods [14].

There are no known specific contraindications for oral contraceptives in GD, unless there is coexisting severe liver disease [9]. Nevertheless, it is recommended to avoid copper IUDs for women with a history of menorrhagia [10]. In addition, the use of contraceptive methods is recommended to both men and women who are under treatment with SRT [11]. For women with GD who desire to plan a pregnancy, SRT must be interrupted 3 months before pregnancy and may be replaced by ERT [15].

### 3.2. Genetic Counseling

GD is an autosomal recessively inherited disorder due to mutations in the *GBA1* gene. All patients with GD should undergo genetic counseling to know the specific risks related to their mutation. For instance, patients homozygous for the p.Asn409Ser mutation (N370S) usually present a milder disease than those who carry others, such as the c.84dup (84GG) [16].

In addition, patients who desire to plan a pregnancy should be able to receive advice on the potential complications during pregnancy and the risk of transmitting her disease to her offspring:

- If the other parent is not a carrier of a *GBA1* mutation, all their children will be carriers, but there is no risk of affected children;
- If the other parent is a carrier of a heterozygous mutation (risk in the general population 0.7–0.8% [17]), 25% of the children will be at risk of being affected by the disease, and 50% will be carriers of the mutation.

Beyond the pre-pregnancy probability of the transmission of the disease to the offspring, it is possible to perform a genetic study in the fetus by taking a sample of chorionic villus or by amniocentesis. Preimplantation diagnosis is also possible to select only embryos that do not carry the mutation, although it must be considered that in vitro fertilization is not a risk-free technique [18].

Full genotyping can be offered to the parents. If the couple carries another pathogenic allelic variant, the predicted genotypes of the offspring and their expected phenotype require in-depth discussions [19].

In addition, parents should be informed that all offspring carrying a mutant *GBA1* allele will have a 4- to 10-fold increased risk of developing Parkinson’s disease [19]. Most patients and their family members who are carriers do not develop Parkinson’s, and those who have been informed about this issue do not change their decision to have children [20]. Even so, the psychological impact must be considered [21].

### 3.3. Monitoring during Pregnancy

The potential complications associated with GD in the context of the physiological changes associated with pregnancy, such as splenic rupture, abnormalities in uterine growth, bone fractures or dysregulation of liver function, have traditionally been a potential contraindication when planning pregnancy in these patients. However, scientific advances in their pathophysiology and improvements in treatment in recent years have changed this approach.

It is important to evaluate the comorbidities of each patient, as well as to monitor the need for nutritional support and the appearance or worsening of organomegalies, bone and hematological manifestations associated with the disease, in order to establish clinical and therapeutic recommendations that allow gestation to develop in conditions comparable to those of the rest of the population [22,23,24,25].

#### 3.3.1. Vitamins and Other Supplementation

Iron deficiency and folic-acid deficiency are frequent findings as gestational age advances, in relation to the increased nutritional requirements of the feto-placental unit. In addition, patients with GD usually present deficiencies of vitamin B12, calcium and vitamin D, which should be adequately supplemented during pregnancy. Although there are no specific recommendations for supplementation, in the case of vitamin D, it is recommended to maintain levels > 32 ng/mL [24,26].

#### 3.3.2. Organomegaly and Bone Manifestations

It is advisable to have a baseline assessment of hepato/splenomegaly to monitor its evolution throughout gestation, usually via an ultrasound.

Once the patient’s bone health has been assessed before the beginning of pregnancy, it is necessary to screen for bone lesions in the pelvic region and lower limbs, as well as to rule out the presence of avascular necrosis of the femoral head. The existence of prosthesis is not a contraindication for delivery, and vaginal delivery is recommended as a preferable option. Likewise, it is necessary to evaluate the loss of bone mass described during pregnancy and lactation to assess the initiation of specific treatment [24,26].

#### 3.3.3. Hematologic Manifestations

The occurrence of hemorrhagic events is one of the symptoms described in approximately 20% of patients with GD. Although these are usually mucocutaneous bleeds, bleeding related to invasive procedures, pregnancy and postpartum have also been reported. Thrombocytopenia is a frequent finding in adult patients with GD, being evidenced in almost 60% of patients with GD type 1, with counts below 60 × 10^9^/L in up to 15% of cases. These figures are variable, depending on the degree of splenomegaly, involvement of megakaryopoiesis by bone marrow infiltration or coexistence of immune pathologies, such as ITP [26,27,28]. In addition, the release of proinflammatory cytokines, such as IL-1, IL-6 or TNF-alpha, together with the accumulation of glucosylceramide and glucosylsphingosine, can produce alterations in platelet functionality and thrombin generation [29,30,31]. Coagulopathies of a very heterogeneous nature have been described, with a predominance of deficiencies of factors XI, II, V, VII and X [28,32]. With these results, the hypothesis of a possible chronic DIC associated with type 1 GD was raised [33].

Due to the existing limitations in the different published studies, there are currently no specific recommendations to guide the follow-ups of these patients. A clinical history is the cornerstone for making a correct differential diagnosis, and allowing the establishment of a specific treatment, given that the exacerbation of baseline thrombocytopenia, alterations in platelet functionality or its appearance during pregnancy can be associated both with the worsening of the disease. Although DIC during pregnancy is rare, it is also important to correlate the clinical picture with laboratory parameters (PT, aPTT, fibrinogen and D-dimer), considering the physiological changes that occur during pregnancy [34,35].

#### 3.3.4. Immunization

A correct vaccination schedule in patients who plan a pregnancy can contribute to the prevention of embryofetopathies, as in the rest of pregnant women. In patients with GD who have undergone splenectomy, due to the increased risk of infection, it is important to review the immunization status prior to pregnancy with the additional recommendation of anti-pneumococcal, anti-Haemophilus influenzae type B and antimeningococcal vaccination [26].

### 3.4. Birth, Postpartum Period and Lactation

The most appropriate approach for the patient will be selected according to the evolution of the pregnancy. To avoid invasive procedures due to the potential risk of bleeding, vaginal delivery is prioritized over cesarean birth. If cesarean birth is required, it is recommended to avoid longitudinal central incisions in the presence of organomegaly. Epidural analgesia is not contraindicated if the patient has no history of bleeding, presents a previous normal blood count and an absence of abnormalities in platelet aggregation or hemostasis studies [24].

Management during the postpartum period will depend on the evolution of the pregnancy, associated comorbidities, and characteristics of delivery. In uncomplicated vaginal delivery, management will be carried out according to usual practice, without specific follow-up recommendations. If surgical intervention is required, monitoring is recommended for the subsequent 24–48 h, considering close follow-up upon discharge in the event of risk factors, usually during the first 6 weeks postpartum.

During the lactation period, the potential appearance of bone complications should be evaluated, due to the transient loss of bone density in 3–7% of the general population, considering, in these patients, limiting their duration to 6 months and evaluating the need for supplementation according to calcium and vitamin D levels [24].

### 3.5. Treatment Recommendations during Pregnancy and Lactation

#### 3.5.1. GD Treatment

When patients with GD are planning a pregnancy, the need for treatment or its modification must be established. It is recommended to perform a dynamic evaluation considering the intercurrent symptoms throughout the pregnancy. The initiation of treatment during pregnancy is recommended in those patients who throughout the pregnancy present symptoms associated with the disease: mainly bone crises, pathological fractures or the worsening of thrombocytopenia with associated bleeding symptoms. The treatment of choice is ERT, with greater experience with Imiglucerase (Cerezyme^®^) compared to Velaglucerase alfa (VPRIV^®^) [12,36]. Treatment with ERT will be prescribed based on pre-pregnancy weight, evaluating cases on an individual basis, according to the clinical evolution of the patient, with the adjustment based on weight gain. The data on treatment with SRT are limited. Preclinical studies reported maternal death in the case of Miglustat (Zavesca^®^) and fetal distress with both Miglustat and Eliglustat (Cerdelga^®^). Thus, SRT is currently discouraged in pregnancy, and it is recommended to discontinue the treatment in the last 3 months. In the Phase 2 and Phase 3 clinical trials of Eliglustat, 18 women had 19 pregnancies, resulting in 14 healthy infants from 13 pregnancies (one set of twins), three elective terminations, one ectopic pregnancy, one miscarriage, and one intrauterine death. If a patient undergoing treatment with Eligustat becomes pregnant without prior planning, Eliglustat discontinuation is recommended [15].

During lactation, GD treatment will follow the same recommendations. If the patient experiences clinical deterioration, lactation interruption or an initiation/adjustment of ERT will be considered depending on the symptoms and the evolution of the patient. In patients with previous bone involvement, no more than 6 months of lactation should be recommended [12,36]. SRT is not indicated in lactation.

#### 3.5.2. Supportive Care

Throughout pregnancy and lactation, the need for nutritional supplements should be assessed on an individual basis according to the symptoms and analytical monitoring. The use of bisphosphonates is discouraged during pregnancy. In those patients undergoing treatment with bisphosphonates, it is recommended to discontinue bisphosphonates 6–12 months prior to pregnancy. Analgesic treatment and other drugs used to treat potential complications throughout pregnancy should follow the standard recommendations [24].

There is little evidence on the management of hemostatic coverage in these patients, and the recommendations established for pregnant patients with congenital or acquired alterations of hemostasis are generally applied, mainly using tranexamic acid for situations of low bleeding risk and platelet transfusion or the use of prothrombin complex concentrates, fresh frozen plasma or fibrinogen, according to the analytical results of each patient, in cases of moderate–severe bleeding risk.

### 3.6. Cancer Screening in the Adult Patient with Gaucher Disease

Although previous studies did not find an increased risk of solid malignancies in patients with GD compared to the general population, this appears to depend on inadequate patient cohorts, and/or differences in the duration and depth of follow-up. Recently, Rosenbloom BE et al. elaborated a large, multicenter, international population with sufficient follow-up times for malignancies to develop. They found a higher risk of malignant neoplasms of liver and kidney cells, breast cancer and melanoma. It seems prudent to monitor for any early signs of hepatic or renal malignancies when examining imaging results during GD1 evaluations; however, the current recommendation is that adult women with GD should undergo the standard screening protocols for breast, colorectal and cervical cancer [37].

### 3.7. Osteoporosis

Osteoporosis and the associated complications such as hip fractures and vertebral prolapse are one of the main complications in GD. Women with bone-density levels of more than 2.5 standard deviations below the young adult reference mean are considered to have osteoporosis. However, since this entity remains asymptomatic until complications develop, it is generally not diagnosed until bone fractures occur [37].

GD is characterized by the presence of “activated macrophages” or Gaucher cells, which infiltrate various tissues, including the bone marrow. These cells induce chronic inflammation, due to the secretion of protease enzymes and the production of inflammatory cytokines and chemokines [38]. Thus, skeletal growth and bone remodeling are altered, and result in the development of osteopenia and osteoporosis, among other alterations [39,40]. Reduced bone density is common in GD. In fact, the most common bone lesions in this entity are medullary infiltration and osteopenia [41,42]. Data from the ICGG Gaucher Registry also indicate that 55% of all patients affected by this lysosomal disease have osteopenia [43].

Sex steroids play an important role in bone growth and in obtaining peak bone mass [44]. Around age 50, women begin to develop substantial bone loss in relation to the estrogen deficiency characteristic of menopause [45]. It is reasonable to assume that in postmenopausal women affected by GD, the concomitance of both factors is associated with a greater severity of loss of bone density, although there are few studies in this regard.

On the other hand, it has been described in women affected by GD that a mean age for the development of menopause is 46.2 years in women without treatment and 49.5 years in women treated with alglucerase/imiglucerase. In the general population, on the other hand, the mean age for menopause is 51 to 52 years [12]. Although more studies are needed, this early onset of menopause will presumably induce a greater loss of bone mineral mass, for which reason screening by dual energy X-ray absorptiometry (DXA)/densitometry seems essential.

Although it seems that ERT induces improvement in the degree of osteopenia, this effect is achieved late after years of treatment. In addition, such osteopenia may transiently worsen immediately after the initiation of therapy [46]. High-dose oral bisphosphonates rapidly increase bone density in patients with GD who are simultaneously treated with ERT [47]; however, it must be considered that one of the possible complications of the use of this antiresorptive therapy is the development of osteonecrosis. The female sex also seems to be related to a higher incidence of this complication.

It is recommended that all post-menopausal women affected by GD undergo periodic densitometries, optimize their intake of dairy products, and proceed to supplementation with vitamin D and calcium if signs of osteopenia are identified. The most chosen treatment strategy for fracture prevention is anti-resorptive therapy with oral bisphosphonates such as alendronate [48]. Anecdotal improvement in bone mineral density and bone architecture commensurate with a reduced incidence of fractures was reported with the bone-anabolic agent teriparatide (human parathyroid hormone (PTH 1–34) in a GD1 patient [49].

Hormone-replacement therapy (HRT) can be offered to peri-menopausal or early post-menopausal women with GD to control moderate to severe menopausal symptoms, after individualizing the risk/benefit of HRT itself [50].

The main complications in each stage are summarized in Table 1.

## 4. Conclusions

Despite the scientific advances achieved in recent years in the pathophysiology of Gaucher disease, there is little evidence of the behavior of the disease symptomatology in the different stages of a woman’s life. The multifactorial etiology of this disease can cause growth retardation and menarche, especially in untreated patients. Alterations in menstrual flow, mainly related to thrombocytopenia or alterations in hemostasis, have also been described in these patients, and the use of oral contraceptives should be considered. Follow-up by a multidisciplinary team is essential during pregnancy in patients with Gaucher disease, first by providing adequate genetic counseling and individualizing the planning of the pregnancy according to the comorbidities present in each patient. It is necessary to guarantee adequate immunization and supplementation, as well as periodically to re-evaluate the existence of organomegaly and bone alterations, to establish safety criteria for delivery. Although there are no specific recommendations to guide the management of alterations in platelet count or functionality or the existence of coagulopathies, these should be monitored to assess the administration of supportive measures. The initiation of treatment with ERT is recommended in those patients who present symptoms related to the disease during pregnancy, and treatment with SRT should be discontinued, due to the limited evidence existing to date, extending these recommendations to the lactation period. Finally, the early onset of menopause, compared to the general population, may accelerate the development of osteopenia in these patients, so performing periodic densitometry is recommended in order to adjust treatment.

## Figures and Tables

**Table 1:** Main risks and recommendations of Gaucher’s disease in women at different stages of life.

Stage of Life		Risks	Recommendations
Childhood		Growth retardation	
Adolescence		Delayed menarche	
Adulthood	General	Abnormal menstrual bleeding Potential development of Parkinson’s disease or neoplasms	Iron supplementation Contraceptive methods Screening protocols
	Pregnancy and lactation	Iron, folic acid, vitamin B12, calcium and vitamin D deficiencies Appearance or worsening of organomegaly Loss of bone mass Hematologic manifestations (thrombocytopenia, rare bleeding disorders) Infections	Supplementation Ultrasound monitoring Consider initiation or adjustment of treatment (ERT ^1^) Consider initiation or adjustment of treatment (ERT ^1^) Consider initiation or adjustment of treatment (ERT ^1^) Hemostatic treatment (antifibrinolytics, platelet transfusion, fresh frozen plasma…) Check immunization
	Menopause	Early onset of menopause Osteoporosis, osteopenia	HRT ^2^ DXA ^3^/densitometry monitoring Consider initiation or adjustment of treatment Support treatment (oral bisphosphonates, vitamin D, and calcium supplementation)

^1^ ERT: enzyme-replacement therapy; ^2^ HRT: hormone-replacement therapy; ^3^ DXA: dual energy X-ray absorptiometry.

## References

[1] Stirnemann J., Belmatoug N., Camou F., Serratrice C., Froissart R., Caillaud C., Levade T., Astudillo L., Serratrice J., Brassier A. (2017). A Review of Gaucher Disease Pathophysiology, Clinical Presentation and Treatments. Int. J. Mol. Sci..

[2] Mistry P.K., Lopez G., Schiffmann R., Barton N.W., Weinreb N.J., Sidransky E. (2017). Gaucher disease: Progress and ongoing challenges. Mol. Genet. Metab..

[3] Torralba-Cabeza M., Morado-Arias M., Pijierro-Amador A., Fernández-Canal M.C., Villarrubia-Espinosa J. (2022). Recommendations for oral treatment for adult patients with type 1 Gaucher disease. Rev. Clin. Esp..

[4] Barney A.M., Danda S., Abraham A., Fouzia N.A., Gowdra A., Abraham S.S.C., Sony M., Das S., Korula S., Mathai S. (2021). Clinicogenetic Profile, Treatment Modalities, and Mortality Predictors of Gaucher Disease: A 15-Year Retrospective Study. Public. Health Genom..

[5] McHugh K., Olsen E.Ø., Vellodi A. (2004). Gaucher disease in children: Radiology of non-central nervous system manifestations. Clin. Radiol..

[6] Mendelsohn E., Meir A., Abrahamov A., Elstein D., Zimran A., Levy-Khademi F. (2018). Growth and final height of children with Gaucher disease: A 15-year follow-up at an Israeli Gaucher center. Blood Cells Mol. Dis..

[7] Kaplan P., Mazur A., Manor O., Charrow J., Esplin J., Gribble T.J., Wappner R.S., Wisch J.S., Weinreb N.J. (1996). Acceleration of retarded growth in children with Gaucher disease after treatment with alglucerase. J. Pediatr..

[8] Kauli R., Zaizov R., Lazar L., Pertzelan A., Laron Z., Galatzer A., Phillip M., Yaniv Y., Cohen I.J. (2000). Delayed growth and puberty in patients with Gaucher disease type 1: Natural history and effect of splenectomy and/or enzyme replacement therapy. Isr. Med. Assoc. J..

[9] Granovsky-Grisaru S., Aboulafia Y., Diamant Y.Z., Horowitz M., Abrahamov A., Zimran A. (1995). Gynecologic and obstetric aspects of Gaucher’s disease: A survey of 53 patients. Am. J. Obstet. Gynecol..

[10] Zimran A., Abrahamov A., Elstein D. (2000). Children with type I Gaucher disease: Growing into adulthood with and without enzyme therapy. Isr. Med. Assoc. J..

[11] Rosenbaum H. (2015). Management of women with Gaucher disease in the reproductive age. Thromb. Res..

[12] Zimran A., Morris E., Mengel E., Kaplan P., Belmatoug N., Hughes D.A., Malinova V., Heitner R., Sobreira E., Mrsić M. (2009). The female Gaucher patient: The impact of enzyme replacement therapy around key reproductive events (menstruation, pregnancy and menopause). Blood Cells Mol. Dis..

[13] Alioto A.G., Gomez R., Moses J., Paternostro J., Packman S., Packman W. (2020). Quality of life and psychological functioning of pediatric and young adult patients with Gaucher disease, type 1. Am. J. Med. Genet. A.

[14] Gold J.I., Gold N.B., DeLeon D.D., Ganetzky R. (2022). Contraceptive use in women with inherited metabolic disorders: A retrospective study and literature review. Orphanet J. Rare Dis..

[15] Lukina E., Balwani M., Belmatoug N., Watman N., Hughes D., Gaemers S.J.M., Foster M.C., Lewis G., Peterschmitt M.J. (2021). Pregnancy outcome in women with Gaucher disease type 1 who had unplanned pregnancies during eliglustat clinical trials. JIMD Rep..

[16] Beutler E., Gelbart T., Kuhl W., Sorge J., West C. (1991). Identification of the second common Jewish Gaucher disease mutation makes possible population-based screening for the heterozygous state. Proc. Natl. Acad. Sci. USA.

[17] Clark L.N., Ross B.M., Wang Y., Mejia-Santana H., Harris J., Louis E.D., Cote L.J., Andrews H., Fahn S., Waters C. (2007). Mutations in the glucocerebrosidase gene are associated with early-onset Parkinson disease. Neurology.

[18] Altarescu G., Renbaum P., Eldar-Geva T., Varshower I., Brooks B., Beeri R., Margalioth E.J., Levy-Lahad E., Elstein D., Zimran A. (2011). Preimplantation genetic diagnosis (PGD) for a treatable disorder: Gaucher disease type 1 as a model. Blood Cells Mol. Dis..

[19] Grabowski G.A., Antommaria A.H.M., Kolodny E.H., Mistry P.K. (2021). Gaucher disease: Basic and translational science needs for more complete therapy and management. Mol. Genet. Metab..

[20] Mulhern M., Bier L., Alcalay R.N., Balwani M. (2018). Patients’ Opinions on Genetic Counseling on the Increased Risk of Parkinson Disease among Gaucher Disease Carriers. J. Genet. Couns..

[21] Verbrugge J., Cook L., Miller M., Rumbaugh M., Schulze J., Heathers L., Wetherill L., Foroud T. (2021). Outcomes of genetic test disclosure and genetic counseling in a large Parkinson’s disease research study. J. Genet. Couns..

[22] Cunningham F.G., Leveno K.J., Bloom S.L., Dashe J.S., Hoffman B.L., Casey B.M., Spong C.Y. (2018). Overview of Obstetrics. Williams Obstetrics.

[23] Queenan J.T., Spong C.Y., Lockwood C. (2012). Queenan’s Management of High-Risk Pregnancy: An Evidence-Based Approach.

[24] Granovsky-Grisaru S., Belmatoug N., vom Dahl S., Mengel E., Morris E., Zimran A. (2011). The management of pregnancy in Gaucher disease. Eur. J. Obstet. Gynecol. Reprod. Biol..

[25] Biegstraaten M., Cox T.M., Belmatoug N., Berger M.G., Collin-Histed T., Vom Dahl S., Di Rocco M., Fraga C., Giona F., Giraldo P. (2018). Management goals for type 1 Gaucher disease: An expert consensus document from the European working group on Gaucher disease. Blood Cells Mol. Dis..

[26] Thomas A.S., Mehta A., Hughes D.A. (2014). Gaucher disease: Haematological presentations and complications. Br. J. Haematol..

[27] Spectre G., Roth B., Ronen G., Rosengarten D., Elstein D., Zimran A., Varon D., Revel-Vilk S. (2011). Platelet adhesion defect in type I Gaucher Disease is associated with a risk of mucosal bleeding. Br. J. Haematol..

[28] Rosenbaum H. (2014). Hemorrhagic aspects of Gaucher disease. Rambam Maimonides Med. J..

[29] Bester J., Pretorius E. (2016). Effects of IL-1β, IL-6 and IL-8 on erythrocytes, platelets and clot viscoelasticity. Sci. Rep..

[30] Page M.J., Bester J., Pretorius E. (2018). Interleukin-12 and its procoagulant effect on erythrocytes, platelets and fibrin(ogen): The lesser known side of inflammation. Br. J. Haematol..

[31] Revel-Vilk S., Naamad M., Frydman D., Freund M.R., Dinur T., Istaiti M., Becker-Cohen M., Falk R., Broide E., Michelson A.D. (2022). Platelet Activation and Reactivity in a Large Cohort of Patients with Gaucher Disease. Thromb. Haemost..

[32] Hollak C.E., Levi M., Berends F., Aerts J.M., van Oers M.H. (1997). Coagulation abnormalities in type 1 Gaucher disease are due to low-grade activation and can be partly restored by enzyme supplementation therapy. Br. J. Haematol..

[33] Serratrice C., Cherin P., Lidove O., Noel E., Masseau A., Leguy-Seguin V., Jaussaud R., Marie I., Lavigne C., Maillot F. (2019). Coagulation Parameters in Adult Patients With Type-1 Gaucher Disease. J. Hematol..

[34] Thachil J., Toh C.H. (2009). Disseminated intravascular coagulation in obstetric disorders and its acute haematological management. Blood Rev..

[35] Simchen M.J., Oz R., Shenkman B., Zimran A., Elstein D., Kenet G. (2011). Impaired platelet function and peripartum bleeding in women with Gaucher disease. Thromb. Haemost..

[36] Lau H., Belmatoug N., Deegan P., Goker-Alpan O., Schwartz I.V.D., Shankar S.P., Panahloo Z., Zimran A. (2018). Reported outcomes of 453 pregnancies in patients with Gaucher disease: An analysis from the Gaucher outcome survey. Blood Cells Mol. Dis..

[37] Rosenbloom B.E., Weinreb N.J., Zimran A., Kacena K.A., Charrow J., Ward E. (2005). Gaucher disease and cancer incidence: A study from the Gaucher Registry. Blood.

[38] Hughes D., Mikosch P., Belmatoug N., Carubbi F., Cox T., Goker-Alpan O., Kindmark A., Mistry P., Poll L., Weinreb N. (2019). Gaucher Disease in Bone: From Pathophysiology to Practice. J. Bone Miner. Res..

[39] Mikosch P., Hughes D. (2010). An overview on bone manifestations in Gaucher disease. Wien. Med. Wochenschr..

[40] Reed M.C., Schiffer C., Heales S., Mehta A.B., Hughes D.A. (2018). Impact of sphingolipids on osteoblast and osteoclast activity in Gaucher disease. Mol. Genet. Metab..

[41] Drelichman G., Linares A., Villalobos J., Cabello J.F., Kerstenetzky M., Kohan R.M., Martins A.M. (2012). Gaucher disease in Latin America. A report from the Gaucher Disease International Registry and the Latin American Group for Gaucher Disease. Medicina.

[42] Rozenfeld P.A., Crivaro A.N., Ormazabal M., Mucci J.M., Bondar C., Delpino M.V. (2021). Unraveling the mystery of Gaucher bone density pathophysiology. Mol. Genet. Metab..

[43] Charrow J., Andersson H.C., Kaplan P., Kolodny E.H., Mistry P., Pastores G., Rosenbloom B.E., Scott C.R., Wappner R.S., Weinreb N.J. (2000). The Gaucher registry: Demographics and disease characteristics of 1698 patients with Gaucher disease. Arch. Intern. Med..

[44] Compston J.E. (2001). Sex steroids and bone. Physiol. Rev..

[45] NIH Consensus Development Panel on Osteoporosis Prevention, Diagnosis, and Therapy (2001). Osteoporosis prevention, diagnosis, and therapy. JAMA.

[46] Cox T.M., Aerts J.M., Belmatoug N., Cappellini M.D., vom Dahl S., Goldblatt J., Grabowski G.A., Hollak C.E., Hwu P., Maas M. (2008). Management of non-neuronopathic Gaucher disease with special reference to pregnancy, splenectomy, bisphosphonate therapy, use of biomarkers and bone disease monitoring. J. Inherit. Metab. Dis..

[47] Wenstrup R.J., Bailey L., Grabowski G.A., Moskovitz J., Oestreich A.E., Wu W., Sun S. (2004). Gaucher disease: Alendronate disodium improves bone mineral density in adults receiving enzyme therapy. Blood.

[48] Giuffrida G., Cappellini M.D., Carubbi F., Di Rocco M., Iolascon G. (2014). Management of bone disease in Gaucher disease type 1: Clinical practice. Adv. Ther..

[49] Khan A., Hanley D.A., McNeil C., Boyd S. (2015). Improvement in Bone Mineral Density and Architecture in a Patient with Gaucher Disease Using Teriparatide. JIMD Rep..

[50] Vigneswaran K., Hamoda H. (2022). Hormone replacement therapy-Current recommendations. Best. Pract. Res. Clin. Obstet. Gynaecol..

